# Functional and structural basis of *E. coli* enolase inhibition by SF2312: a mimic of the carbanion intermediate

**DOI:** 10.1038/s41598-019-53301-3

**Published:** 2019-11-19

**Authors:** Jolanta Krucinska, Michael N. Lombardo, Heidi Erlandsen, Akram Hazeen, Searle S. Duay, Jason G. Pattis, Victoria L. Robinson, Eric R. May, Dennis L. Wright

**Affiliations:** 1 Department of Pharmaceutical Sciences, University of Connecticut, 69 North Eagleville Road, Storrs, Connecticut 06269 United States; 2 Center for Open Research Resources & Equipment (COR2E), University of Connecticut, 91 North Eagleville Road, Storrs, Connecticut 06269 United States; 3 Department of Molecular and Cellular Biology, University of Connecticut, 91 North Eagleville Road, Storrs, Connecticut 06269 United States; 4 Department of Chemistry, University of Connecticut, 55 North Eagleville Road, Storrs, Connecticut 06269 United States

**Keywords:** Biophysics, Drug discovery, Structural biology

## Abstract

Many years ago, the natural secondary metabolite SF2312, produced by the actinomycete *Micromonospora*, was reported to display broad spectrum antibacterial properties against both Gram-positive and Gram-negative bacteria. Recent studies have revealed that SF2312, a natural phosphonic acid, functions as a potent inhibitor of human enolase. The mechanism of SF2312 inhibition of bacterial enolase and its role in bacterial growth and reproduction, however, have remained elusive. In this work, we detail a structural analysis of *E. coli* enolase bound to both SF2312 and its oxidized imide-form. Our studies support a model in which SF2312 acts as an analog of a high energy intermediate formed during the catalytic process. Biochemical, biophysical, computational and kinetic characterization of these compounds confirm that altering features characteristic of a putative carbanion (enolate) intermediate significantly reduces the potency of enzyme inhibition. When SF2312 is combined with fosfomycin in the presence of glucose-6 phosphate, significant synergy is observed. This suggests the two agents could be used as a potent combination, targeting distinct cellular mechanism for the treatment of bacterial infections. Together, our studies rationalize the structure-activity relationships for these phosphonates and validate enolase as a promising target for antibiotic discovery.

## Introduction

The ever-present and rapidly growing threat of antibiotic-resistant bacteria, coupled with a significant void in antibiotic drug discovery, underscores the need for the continuing identification of new bacterial targets for the development of therapies with a novel mode of action. Either alone or in combination, such therapeutics are valuable as they are unlikely to display cross-resistance to commonly used agents and they provide a powerful weapon to the antibiotic armamentarium. The glycolytic pathway, a series of metabolic reactions catalyzed by multiple enzymes and enzyme complexes, has garnered substantial attention as a potentially rich source of drug targets for multiple indications. Glycolysis is not only crucial for energy utilization but also results in the synthesis of vital building blocks required for cell division. Bacterial enolase, a metalloenzyme involved in carbon metabolism, is essential to bacterial glycolysis and the generation of metabolites used in several bacterial cell processes. Despite their critical nature, there has been little effort directed towards developing antibiotics targeting the glycolytic enzymes. We are excited by the opportunity presented by bacterial enolase as a new potential target for antibacterial development.

Enolase, a multifunctional metalloenzyme, catalyzes the dehydration of 2-phosphoglycerate (2-PGA) to phosphoenolpyruvate (PEP) through the intermediacy of a stabilized carbanion. Glycolysis is completed when PEP is converted into pyruvate, producing one molecule of adenosine triphosphate (ATP) in the process through substrate-level phosphorylation. Prokaryotes express a single isoform of enolase which, in addition to acting as a key mediator of energy production, appears to be involved in the overall infection process - enhancing bacterial resistance, invasiveness, and tissue damage^1^. For example, in both *Streptococcus canis* and *Streptococcus pneumoniae*, enolase binds plasminogen, facilitating entry into the host cell, dissemination throughout the host and evasion of immune system^1,2^. These findings suggest that inhibiting this essential enzyme may have direct effects on both bacterial viability and pathogenicity.

Mammals, like bacteria, employ enolase in their glycolytic processes. How enolase inhibitors would impact mammalian cells must, therefore, also be considered. Unlike bacteria, the human genome encodes four distinct isoforms of enolase that are constitutively expressed but are largely tissue specific. Enolase 1 (ENO1), the predominant isoform, is ubiquitously expressed in adult human tissues including the liver, brain, kidney, and spleen. Enolase 2 (ENO2) is expressed in neuronal cells while enolase 3 (ENO3) is localized to muscle tissue and the recently-discovered enolase 4 (ENO4) is specific to the male reproductive cells^3^. While the individual isoforms are involved in numerous non-glycolytic biological processes, the expression of multiple human isoforms primarily provides redundancy for their role in glycolysis^4^. The importance of this redundancy is highlighted by certain lineages of glioblastoma where the gene encoding ENO1 is collaterally deleted along with tumor suppressor genes during a translocation event. Importantly, the viability of the malignant cells is maintained through ENO2 expression^4,5^. Furthermore, it was shown that broad inhibition of enolase had minimal toxicity in ENO1-intact glioma cells and normal human astrocytes while being *lethal* to the ENO-1 deficient cells, an appropriate setting for employing a ‘synthetic lethal’ strategy. Beyond the glycolytic redundancy, there are structural differences between the bacterial and mammalian enolase enzymes that could be exploited to enhance target-level selectivity when designing an enolase-targeting antibiotic. Recently, we identified a series of antimicrobial tropolones that target the *E. coli* enolase, supporting the hypothesis that inhibition of enolase can be an effective strategy for the development of new antibacterial agents^6^. Further support for this approach was found with the naturally occurring antibiotic SF2312. Isolated several decades ago, the mechanism of action of this natural phosphonic acid remained elusive^7^. Upon the discovery that SF2312 has anticancer activity in ENO1-deleted glioblastoma via ENO2 inhibition^5^, we became interested in examining this natural product to determine whether or not its antibiotic effects were mediated through the bacterial enolase, and, if so, what interactions were essential to the inhibitory process.

**Scheme 1:**
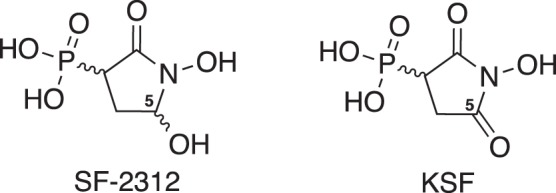
Structures of SF2312 and KSF.

Even though multiple structures of various eukaryotic enolases have been reported, few structures of *E. coli* enolase have been made available. Herein, we present novel crystal structures of *E. coli* enolase in complex with SF2312 and its oxidized derivative, KSF where the C5 hemiaminal is oxidized to an imide carbonyl unit (Scheme 1). This latter modification of SF2312 was implemented to determine the role of the hemiaminal in the active site of *E. coli* enolase. Of particular interest was the possibility that SF2312 could bind irreversibly to the enzyme by reaction of the hemiaminal (or the open chain aldehyde tautomer) with the nucleophilic Lys341 residue. Oxidation of this functionality would be expected to eliminate this potential site of electrophilic reactivity. Considering the importance of metal ions to enolase activity, we used metadynamics methods for molecular dynamics (MD) to conduct molecular simulations on the enzyme complexed with both ligands, in the context of the catalytic Mg2. We constructed free energy landscapes of unbinding of SF2312 and KSF from enolase to establish the key determinants of the metal-dependent transition between ‘bound’ and ‘unbound’ state of both ligand complexes. Finally, the antibacterial properties of SF2312 were tested *in vitro* against a panel of reference bacterial strains (*A. baumannii*, *E. coli*, *P. aeruginosa*, and *S. aureus*). The minimum inhibitory concentrations (MICs) of the SF2312 alone and in combination with fosfomycin were determined in presence of glucose-6-phosphate to establish if these agents could act synergistically to further increase their antimicrobial efficacy. Collectively, the structural studies, corroborated by biochemical, molecular dynamics simulations and microbiological data indicate that SF2312 is a potent inhibitor of *E. coli* enolase, providing a paradigm for further optimization of its antibacterial properties as a combinatorial therapy with phosphonic acid antibiotics such as fosfomycin.

## Results and Discussion

### Structures of *E. coli* enolase bound with SF2312 and KSF

Crystal structures of enolase bound with SF2312 or KSF were generated through co-crystallization of the recombinant *E. coli* enolase with an excess amount of inhibitor and Mg^2+^ to ensure the stability of the protein-ligand complex. The crystals diffracted to 2.2 Å and 2.5 Å, respectively, belong to the space group P2_1_2_1_2_1_ and were refined to an R_free_ of 21.6% and 25.6% (Table 1). In both structures, *E. coli* enolase formed three dimers in a hexameric arrangement that interact through a N-terminal T7-tag. While there is full density for SF2312 ligand in all six monomers, KSF is present in only three out of the six active sites. Interestingly, in the ligand unbound sites we instead observed density for tartrate, which is a component of the crystallization medium.

**Table 1:** Data collection and structure refinement statistics for EcENO:SF2312 and EcENO: KSF.

	SF2312: ecEnolase	KSF: ecEnolase
**Data collection**		
Beam line	SSRL: 9-2	SSRL: 14-1
Wavelength (Å)	0.9792	1.19499
Space group	P2_1_2_1_2_1_	P2_1_2_1_2_1_
Cell parameters		
a, b, c (Å)	104.75,142.07, 207.28	104.18, 143.11, 206.68
α, β, γ (º)	90, 90, 90	90, 90, 90
Resolution range (Å)^#^	93.53–2.24 (2.34–2.24)	93.03–2.57 (2.62–2.57)
Total observations	1,517,907 (189,714)	187,179 (8683)
Unique observations	147,816 (17,855)	97,620 (4421)
Completeness (%)	99.8 (95.8)	99.4 (92.9)
Mean *I/σI*	18.1 (5.5)	5.9 (0.7)
Multiplicity	10.3 (10.6)	1.9 (2.0)
R_meas_ (%)	0.098 (0.467)	0.106 (0.560)
CC(1/2)	0.999 (0.958)	0.984 (0.791)
**Refinement**		
Resolution range (Å)	207.28–2.24	93.20–2.57
R_work_/R_free_ (%)	16.3/21.6	19.1/25.6
RMSD bond length (Å)	0.0174	0.0080
RMSD bond angles (º)	1.812	1.599
Ramachandran plot		
Most favored	97.2%	94.44%
Allowed	2.4%	4.86%
Disallowed	0.4%	0.7%
Average B factor (Å^2^)		
Main chain	36.2	49.36
Side chain	41.5	52.84
Ligand	47.7	71.07
Water	32.0	34.53
No. of atoms in model		
Protein	19,500	19325
Ligands	174	151
Waters	520	248
PDB identity code	6D3Q	6NPF

**Figure 1:**
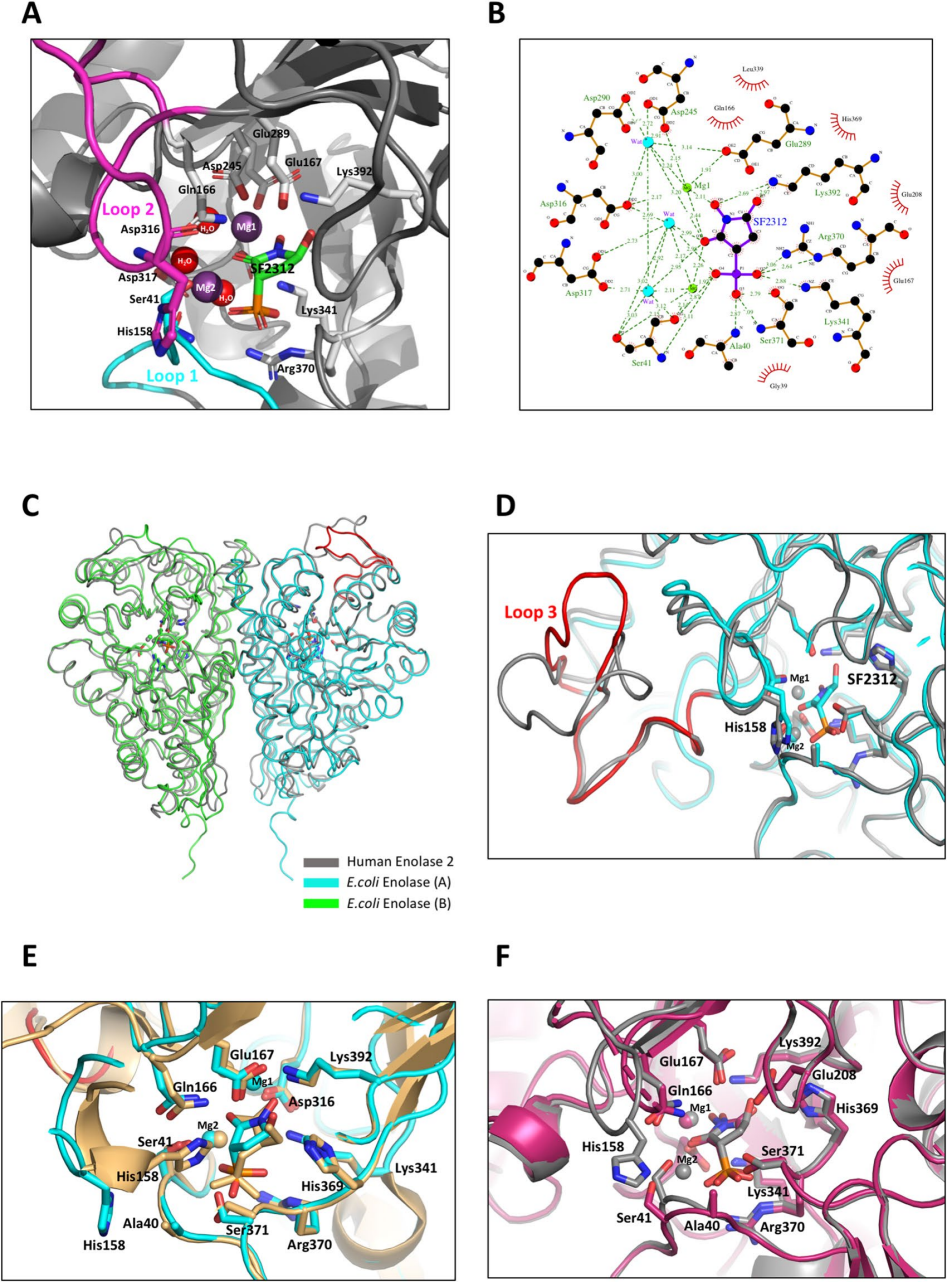
(**A**) Close-up of active site of *E. coli* enolase showing residues interacting with the SF2312 inhibitor. Loop 1 is colored cyan and Loop 2 is colored magenta. (**B**) Ligplot schematics of interactions at active site in the SF2312-bound *E. coli* structure. (**C**) Superposition of the *E. coli* and human enolase dimer structures complexed with SF2312. (**D**) Close-up of active site showing Loop 3 (residues 248–268) in red for the *E. coli* structure. (**E**) Active site representation of the superposition of *E. coli* enolase in complex with SF2312 (cyan) and substrate 2-PGA (sand; PDB id code 6BFY). (**F**) Active site representation of the superposition of *E. coli* enolase (chain F) in complex with KSF (magenta) onto *E. coli* enolase with SF2312 (gray).

The binding mode of SF2312 in *E. coli* enolase structure is similar to that of human ENO2, the only other reported crystal structure of SF2312 bound to a protein^5^ (see Fig. 1). The hydroxamic acid mimics the carboxylate of 2-PGA coordinating with Mg1 as well as hydrogen bonding with Gln166, Asp316, and Lys392. The amide of Ala40 and Ser371 work in concert with His158, Arg370 and Mg2 to neutralize the phosphonate group of 2-PGA^6,8,9^. The same interactions are observed between the phosphonate group of SF2312 and the active site residues, however an additional interaction with Lys341is formed. The hydroxyl group of SF2312, analogous to the C-3-OH of 2-PGA, makes an extensive series of hydrogen bonds with Glu167, Lys392, and His369. No formation of Schiff base-like adducts between Lys341 and the hemiaminal moiety of SF2312 were observed in this structure.

A comparison of the binary structures of *E. coli* enolase with KSF and SF2312 reveals significant structural differences between the two complexes. Unlike in the *E. coli* enolase:SF2312 structure, Loop 2 (residues 154–165) and Loop 3 (residues 248–268) are disordered and Mg2 is absent from the active site of the enolase:KSF complex. The three monomers containing KSF all adopt the same conformation with an average RMSD of 0.29, however in chain E, the pyrrolidine is flipped ~135° compared to chains D and F where KSF adopts a similar orientation to SF2312. The planarity of KSF appears to allow binding in both orientations positioning the hydroxamic acid to coordinate Mg1 and overlaying the phosphonate group (see Fig. 1F). Chain D, which had the best density for KSF, was used for structural analysis.

Similar to SF2312, the bidentate hydroxamate of KSF completes the octahedral coordination of Mg1 while hydrogen bonding with the charged side chains of Gln166, Asp316, and Lys392. The phosphonate group of KSF is also within hydrogen bonding distance of Arg370, Ser371, Ser41, and Lys341. Due to the displacement of Loop 2 and 3 and the absence of the catalytic Mg2 in the active site, the overall complex appears considerably weaker in comparison to SF2312. Interestingly, when the hydroxyl group of SF2312 is oxidized to a carbonyl, as in KSF, it still maintains interactions with Glu167, His369, and Lys 392, and forms a potential hydrogen bond with Glu208. This observation points to a critical role for the hydrogen bond donor in inhibition of the enzyme that renders these two very similar compounds quite distinct in their modes of inhibition.

The catalytic cycle of enolase, especially yeast and lobster enolase, has been a subject of many studies and is well characterized^8–10^. The crystal structures of *E. coli* enolase with 2-PGA and PEP that we recently reported^6^, support the hypothesis that enolase undergoes a similar two-step catalytic process where conversion of 2-PGA to PEP is initiated by the removal of a proton from C-2 yielding a carbanion (enediolate) intermediate, and the reaction is ultimately completed by β-elimination of hydroxide^11^.

The active site of enolase is a highly charged mixture of basic and acidic residues, creating an ideal environment to lower the pKa of C-2 and facilitate deprotonation^11^. Upon binding, the 2-PGA carboxyl group forms hydrogen bonds with Gln166, Lys392, and Mg1. Loop 1 (residues 38–46) moves ~11 Å such that Ser41 makes additional contacts with 2-PGA and Gln166 of Loop 2 and coordinates with the catalytic Mg2. The interplay between Loop 2 and 3 is critical as it brings His158 within range to donate a proton to the phosphate of 2-PGA. Together with a close proximity to Arg370 and the presence of Mg2, the phosphate group is over-neutralized which subsequently leads to a lowering of the pKa of carbon into the physiological range.

Moreover, our structures of *E. coli* enolase agree with previously published work on the catalytic mechanism, as Lys341 appears to serve as the catalytic base abstracting the proton of C-2 (2-PGA) to give the carbanion intermediate^12^. The microenvironment of Lys341 serves as the primary structural evidence for Lys341 acting as the catalytic base. In the unbound structure with Mg1^6^, the predicted pKa of Lys341 is 7.56, indicating that Lys341 is likely unprotonated in the crystal structure (pH = 6.0). Upon 2-PGA binding, Lys341 hydrogen bonds with Asp316 (bidentate) as well as a water molecule coordinated to Mg2 as is evident in the bound structure of *E. coli* enolase with 2-PGA (PDB ID: 6BFY). Following conversion to PEP, Lys341 appears to be protonated with a predicted pKa of 8.9 and forming a third hydrogen bond with an oxygen of the phosphate group.

### SF2312 is an analogue of the carbanion intermediate

A comparison of the structures of *E. coli* enolase bound to SF2312, KSF and 2-PGA reveals features critical for potency. Conformational changes and significant differences in the binding mode of SF2312 relative to the substrate in the enzyme active site, suggest it is not merely a substrate analog. The most notable difference is positioning of Loop 2; specifically, the Cα of His158 is shifted 3.4 Å away from the active site (see Fig. 1E). His158 is important to the hydrolytic reaction in that it protonates the phosphate of 2-PGA to allow for an electron withdrawing effect from C-2^13^. Lys341 abstracts the proton from C-2 forming the carbanion intermediate and forms a hydrogen bond with the phosphate, thereby releasing His158 from its hydrogen bond^13^. The structure of SF2312 reveals a similar network of interactions. Additionally, the hydroxamic acid of SF2312 serves as a more stable metal chelating group forming a 2.1 Å bond with Mg1, approximately 1 Å tighter than 2-PGA. This interaction better represents the more concentrated negative charge of the enediolate intermediate rather than the carboxylate. Moreover, a 5-membered ring chelating complex formed between the hydroxamic acid of SF2312 and Mg1 as compared to only a 4-membered carboxylate coordination in 2-PGA, reflects a more stable and tighter binding event. While SF2312 exists as a racemic mixture in solution due to stereocenters at the 3- and 5-positions, it is noteworthy that in our complex structure with *E. coli* enolase, only the 3 S,5 S configuration is observed. This is further confirmed in the recent work which describes the human ENO2 enolase in complex with the active S,S-enantiomer form of SF2312^14^.

As there was no evidence that the hemiaminal functionality of SF2312 was involved in covalent modification of the active site, it was anticipated that KSF may maintain a similar inhibition profile to SF2312 as it retains hydrogen bond accepting capabilities at the O-5 position. Interestingly, structural data revealed that an additional interaction is even possible with Glu208. However, the enzyme adopted more of an “open” overall conformation, being more representative of the product-bound rather than the intermediate-bound state (PDB 6BFZ). Importantly, the change in hybridization of C5 from sp^3^ tetrahedral geometry in SF2312 to sp^2^ trigonal planar geometry in KSF more accurately reflects the geometry of the PEP product rather than the anionic intermediate. The downstream effect of this observation manifests in a very significant reorientation of the phosphate group which no longer interacts with His158 and completely destabilizes Loops 2 and 3. This major conformational change is reflected in over 400-fold loss in activity between SF2312 and KSF (SF2312 IC_50_: 18.4 ± 2.84 nM, KSF IC_50_: 7.67 ± 0.38 µM).

### Kinetic characterization of *E. coli* enolase activity and inhibition

**Figure 2:**
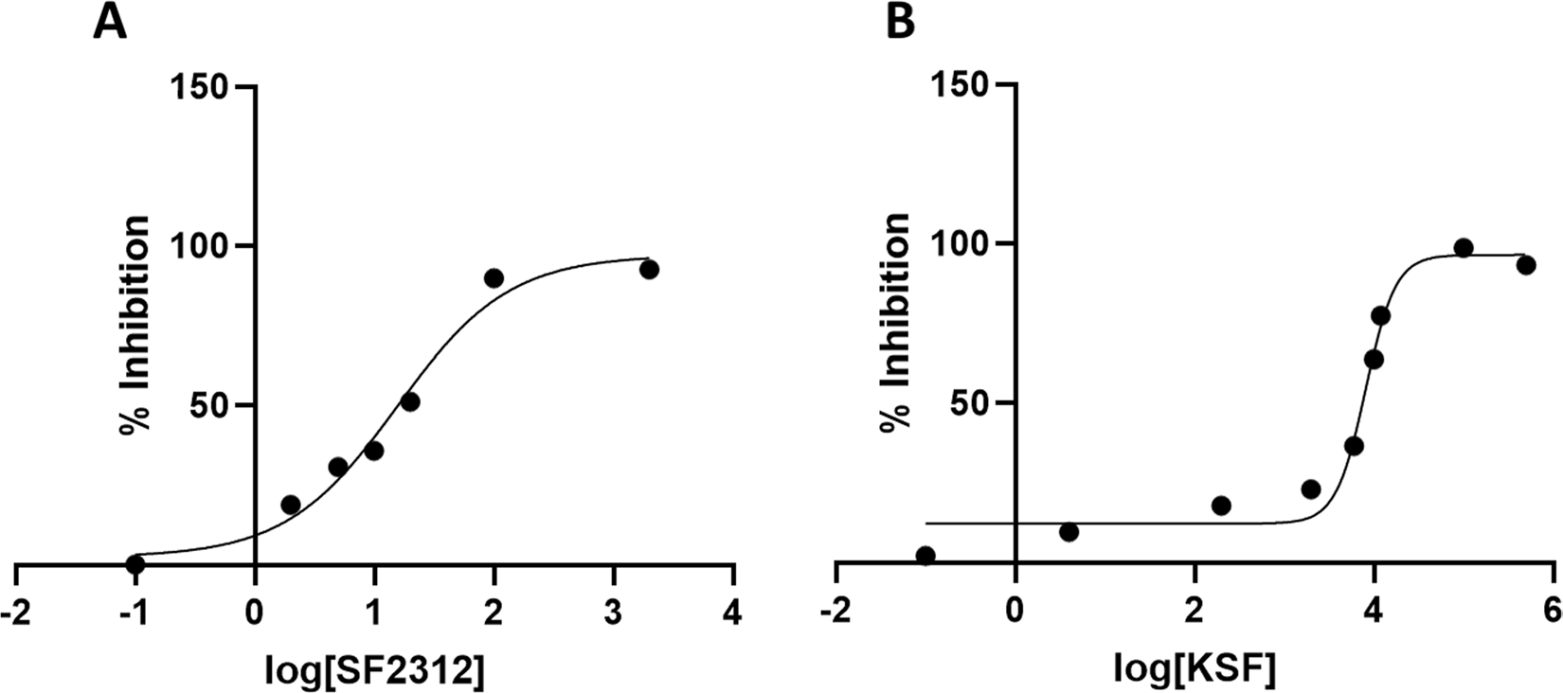
A dose response curve for the recombinant enolase enzyme (40 nM) in the presence of 2.0 nM to 2.0 μM SF2312 (**A**) and 4.0 nM to 100 μM KSF (**B**). The IC_50_ and Ki values of SF2312 and KSF were 18.4 nM (Ki = 3.4 nM) and 7.7 μM (1.4 μM) respectively.

The *K*_M_ of *E. coli* enolase for 2-PGA were determined from measurements of initial rates of the reactions using the non-linear regression analysis (GraphPad Prism8) (Fig. 2). The enzyme displayed classical Michaelis–Menten kinetics. The *K*_M_ value for 2-PGA and *V*_max_ value were 113 μM and 19.56 ± 2.4 μM min^−1^ mg^−1^ at 25 °C, respectively. This leads to a calculated k_cat_ of 8.5 s^−1^ and a catalytic efficiency (k_cat_/K_M_) of 0.07 µM^−1^ s^−1^. Except for *T. maritima* and *T.aquaticus*, the substrate binding affinity of *E. coli* enolase is similar to that found in other sources such as *S.cerevisiae*, *C.albicans*, *T.brucei* and *P.falciparum*^15^.

To assess the relative affinity of SF2312 and KSF for enolase, the inhibitory constant, K_i_, was determined from IC_50_ values using the Cheng-Prusoff equation and K_M_ values. SF2312 exerted potent inhibition on enolase, with K_i_ values of 3.4 nM. However, the activity of KSF against the recombinant enzyme was significantly weaker, with K_i_ = 1.4 μM.

### Thermal unfolding studies of enolase bound to the inhibitors

**Figure 3:**
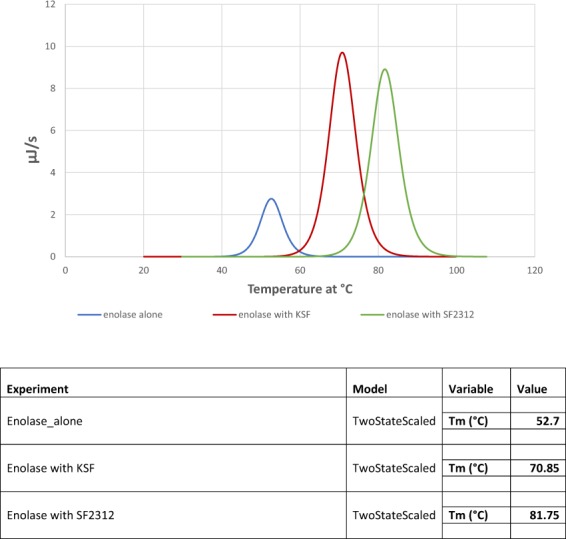
DSC thermogram of enolase (apo; blue), with SF2312 inhibitor (green) and KSF (red).

We used differential scanning calorimetry to analyze the direct *in vitro* binding of SF2312 and KSF to recombinant *E. coli* enolase (see Fig. 3). In the absence of inhibitors, *E. coli* enolase showed thermal denaturation at ∼53 °C. Upon overnight treatment with SF2312, the protein thermal melting point was ~82 °C, a shift of ∼29 °C. Interestingly, treatment with an equivalent concentration of KSF resulted in a more modest shift of ∼18 °C, indicating a less stable inhibitory complex. The larger shift in thermal melting point seen with SF2312 compared to that of KSF correlates well with our enzyme inhibition data in which SF2312 demonstrates a nanomolar IC_50_ compared to micromolar levels for KSF. The data implicitly suggests that the increased potency of SF2312 is due to a tighter, more stable protein-small molecule complex.

### Residence time assay

Although the crystal structure suggested that there was no irreversible interaction between enolase and SF2312, we wanted to probe this same possibility in solution by measuring the inhibitor's residence time. Determination of inhibitor residence times (t_R_), the time that a drug remains bound to its target before dissociating, can provide more detail into the pharmacodynamic profiles of antibiotics. In general, longer engagement with the target can result in improved efficacy, as the extended contact between drug and enzyme translates into a more efficient blockade of the metabolic pathway.

To assess the reversibility of enolase inhibition by SF2312 and its analog, the dissociation rates of the inhibitors were measured using the jump-dilution method described by Copeland^16^. Prior to the jump-dilution measurements, we first determined the optimal enzyme concentration for the assay (0.4 nM after 100x dilution) that would provide a robust signal. Next, to allow complexes to fully form, the recombinant tagged enolase at 40 nM concentration was preincubated with SF2312 (200 nM) and KSF (80 µM) which corresponds to around ten times the IC_50_ values of each ligand, respectively. The control sample (without inhibitor) was also prepared. After incubation for 16 hours, both mixtures were diluted 200-fold by adding 2.5 μL of the incubated sample into 497.5 µl of dilution buffer containing saturating amount of the substrate (500 µM 2-PGA). The recovery of enolase activity was monitored kinetically every 1 minute for 1 hour. After converting the raw data to product formation (PEP), reaction progress curves were fitted to an integrated rate equation:

$$\lceil P\rceil ={V}_{s}t+\frac{{V}_{i}-{V}_{s}}{{k}_{obs}}(1-{e}^{-{k}_{obs}t})$$

where V_i_ is the initial velocity observed in the presence of the inhibitor (fully inhibited) and V_s_ is the steady-state velocity after dilution (uninhibited), k_obs_ is the apparent first-order rate constant for the transition from *v*_*i*_ to *v*_*s*_, and *t* is time of reaction. Under conditions of minimal inhibitor rebinding the observed rate constant, k_obs_ approximates the dissociation rate constant (k_off_) of the enzyme–inhibitor complex, and therefore allows inhibitor residence time to be estimated as 1/k_obs_.

**Figure 4:**
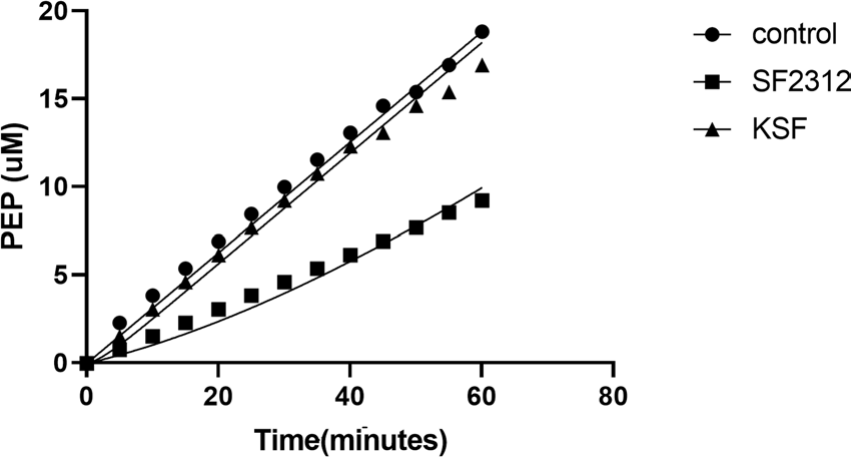
Residence time of SF2312 and KSF with enolase plotted as a function of PEP formed.

From the plot of product (PEP) formation as a function of time (Fig. 4) it is suggestive that both ligands exhibit a reversible behavior. The residence time for SF2312 was 67 minutes while the recovery of enzymatic activity for KSF was much shorter, with a residence time of 2.7 minutes. Consequently, the progress curve for fast dissociating ligand like KSF was linear with a slope nearly equal to the slope of the uninhibited enolase sample. In contrast, the SF2312 treated sample yielded a sufficient curvature in the time course after rapid dilution. Taken together with our structural data it is plausible that the slow dissociation of SF2312 is coupled with ordering of the catalytic loop (Loop 2) that covers the entrance to the binding pocket and thereby locks the inhibitor into the cavity and increases its residence time. In summary, we observed a correlation between enzymatic inhibitory activity of SF2312 and KSF on enolase and the time they remain bound to the target.

### Metadynamics simulations

**Figure 5:**
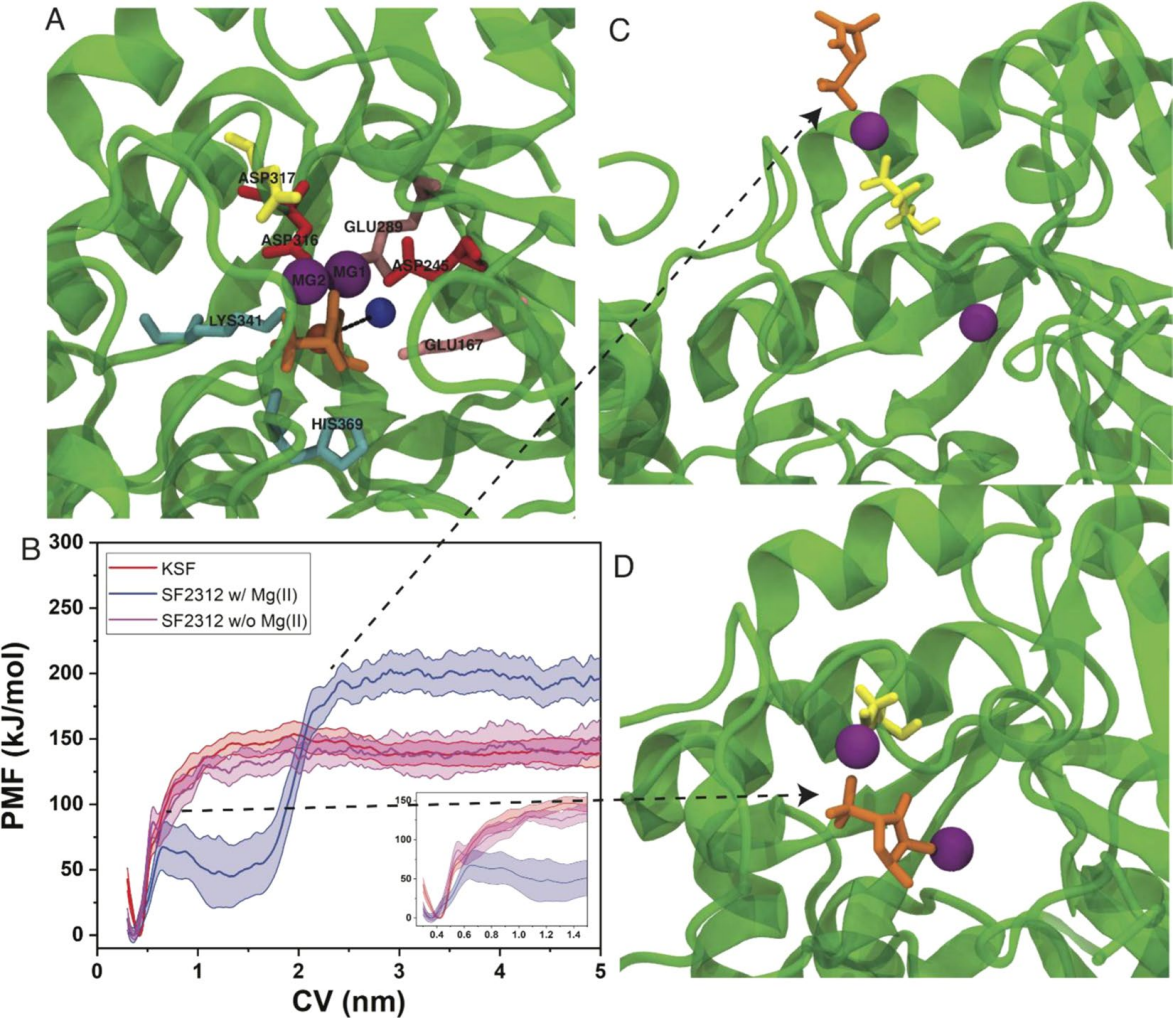
(**A**) The CV for metadynamics is represented by the distance (black line) between the COM of the ligand (orange sphere) and the COM of the labeled residues (blue sphere), excluding residue ASP317. (**B**) Relative free energy profiles of unbinding for KSF, SF2312 with Mg2, and SF2312 without Mg2, insert figure shows a zoomed in PMF in the range from 0.3–1.5 nm in the CV. The bound configuration of SF2312 is shown in (**A**), while representative structures of the first and second energy barriers are shown in panels (D,C), respectively. In panels (A,C,D) the Mg ions are represented as purple spheres, Asp317 is shown in yellow and SF2312 is shown in orange.

The relative free energies of unbinding of SF2312 and KSF from enolase were obtained by running frequency adaptive metadynamics simulations. To generate ligand unbinding trajectories, the simulations were biased along a collective variable (CV), which describes the ligand dissociation coordinate. A single CV was chosen which was the distance between the ligand center-of-mass (COM) and the collective COM of six residues (E167, D245, E289, D316, K341, H369) on the β-barrel of enolase, below the binding site (Fig. 5A). From each unbinding trajectory a free energy profile (potential of mean force, PMF) is obtained and the individual PMFs are then averaged (Fig. 5B). It was observed that the PMF profiles were qualitatively different for SF2312 and KSF unbinding. The KSF profile indicates a one-step process with a single energy barrier around 0.6 nm in the CV and barrier height around 65 kJ/mol. The SF2312 PMF also displays an energy barrier in a similar location and of similar height to the KSF barrier, however SF2312 displays a large basin following the first barrier. The basin extends out to approximately 1.7 nm in the CV and the height of the barrier is considerably larger than first barrier (> 100 kJ/mol). From these PMFs it can be inferred that the rate-limiting step in the dissociation of SF2313 would be the second energy barrier and would indicate a longer residence time for SF2312 than the KSF.

Interestingly, from the trajectories of the SF2312 simulations, it was observed that Mg2 dissociates concomitantly with the inhibitor (Fig. S1). The first energy barrier is characterized by loop opening, and the ligand and Mg2 becoming more exposed to the solvent (Fig. 5D). In all eleven trials, we observed that the Mg2 and Asp317 have sustained interactions in the metastable state and the second energy barrier appears to be arising from breaking this contact to fully release SF2312 from the protein (Fig. 5C). We quantified which residues come into contact with Mg2 during the simulation, using a 4 Å cutoff distance, and find that Asp317 has the most consistent interactions and is the last residue to be in contact with Mg2 before dissociation (Fig. S2). To test the importance of Mg2 in stabilizing the interaction of SF2312 with enolase, we ran another set of metadynamics simulations with Mg2 removed from the system. We find that the PMF for SF2313 without Mg2, more closely resembles the KSF PMF and does not display the metastable state and second energy barrier.

From the metadynamics simulation time of unbinding (see Fig. S3 and Table S1), we observe KSF has the shortest characteristic time of unbinding, followed by SF2312 without Mg2, and SF2312 with Mg2 has the longest characteristic unbinding time. Relating these simulation times directly to experimental residences times would require a rescaling procedure^17^ or a different set of simulation parameters (non well-tempered)^18^. However, the metadynamics unbinding times are qualitatively consistent with the experimental residence times and support that SF2312 has a greater residence time in enolase than that of KSF.

### Evaluation of the antibiotic activity of SF2312

The antibacterial activity of SF2312 was evaluated against a small panel of priority pathogens via minimum inhibitory concentration (MIC) determination for *Acinetobacter baumannii*, *Escherichia coli*, *Pseudomonas aeruginosa*, and *Staphylococcus aureus*. Initial results showed SF2312 had an MIC of 400 µg/mL, despite potent *in vitro* enzyme inhibition (see Table 2). We noted that fosfomycin, an antibiotic often used to treat uncomplicated urinary tract infections, contains a highly charged phosphonate group, similar to SF2312, and must be actively taken up into bacterial cells because of the high polarity^19^. In *E. coli* and *S. aureus*, two inducible transmembrane proteins, glycerol-3-phosphate transporter (GlpT) and glucose-6-phosphate transporter (UhpT), are known to facilitate the active transport of fosfomycin^20,21^. The expression of these protein systems is induced by presence of their substrates, and as such, fosfomycin requires media supplemented with glucose-6-phosphate to observe significant antibacterial activity in *in vitro* assays^19^. When media was supplemented with 25 µg/mL of glucose-6-phosphate, there was a significant improvement in activity against *E. coli* and *S. aureus* with MIC values of 100 and 20 μg/mL respectively. However, there was no significant change in the MIC values against *A. baumannii* and *P. aeruginosa* (see Table 2). It is well-established that *P. aeruginosa* lacks a homologue for UhpT, thus fosfomycin activity relies solely on GlpT for intracellular accumulation into this pathogen^22^. This suggests that SF2312 is exclusively transported by UhpT. Conversely, *A. baumannii* is intrinsically resistant to fosfomycin. Considering a limited amount of data available, it appears that drug efflux and peptidoglycan recycling contribute to resistance, not an impaired fosfomycin uptake, providing no insight into fosfomycin resistance^23,24^.

**Table 2:** Antibacterial Activity of SF2312 and fosfomycin in CAHM media with and without 25 μg/mL of Glucose-6-Phosphate (represented as MIC in μg/mL).

	Without Glucose-6-Phosphate in the media		With Glucose-6-Phosphate in the media	
Microorganism	SF2312	fosfomycin	SF2312	fosfomycin
*E. coli ATCC 25922*	400	>10	100	1.25
*A. baumannii ATCC 19606*	>400	>20	>400	>20
*S. aureus ATCC 43300*	100	20	20	2.5
*P. aeruginosa ATCC 27853*	50	5	50	5

It should be noted that the target of fosfomycin, UDP-N-acetylglucosamine enolpyruvyl transferase (MurA), acts immediately downstream of enolase utilizing PEP in the first step of peptidoglycan synthesis. Since targeting two sequential enzymes in the same metabolic pathway can be a powerful synergistic mechanism for two antibiotics, an evidence for synergy between SF2312 and fosfomycin was assessed by the checkerboard method^25^. When co-administered, the *E. coli* MIC for SF2312 and fosfomycin dropped a maximum 16-fold and 4-fold, respectively, and the drug combination was deemed synergistic, defined as a fractional inhibitory concentration (FIC) index less than 0.5 (Table 3)^26^. The observed synergy supports the notion that the antibacterial activity of SF2312 is due to the inhibition of enolase since, fosfomycin is known to have minimal to no effect on the enzymes utilizing PEP, such as enolase^27^.

**Table 3:** Inhibitory activity of combinations of SF2312 with fosfomycin on the growth of live bacteria, *E. coli* ATCC 25922.

MIC (μg/mL) SF2312	MIC (μg/mL) Fosfomycin	FIC SF2312 SF2312	FIC Fosfomycin Fosfomycin	*FICI
100	0	—	—	—
25	0.156	0.25	0.1248	**0.3748**
12.5	0.312	0.125	0.2496	**0.3746**
6.25	0.312	0.0625	0.2496	**0.3121**
3.12	0.625	0.0312	0.5	0.5312
0	1.25	—	—	—

*The fractional inhibitory index (FICI) is calculated as follows:

FICI = (MIC of SF2312 in the presence of FOS/MIC of SF2312 alone) + (MIC of FOS in the presence of SF2312/MIC FOS alone).

A FICI of ≤0.5 indicates synergism, a value of 0.5 to 4 indicates no interaction, and a value of >4.0 indicates antagonism.

### Comparison with human enolase isoforms: approaches to selectivity

**Figure 6:**
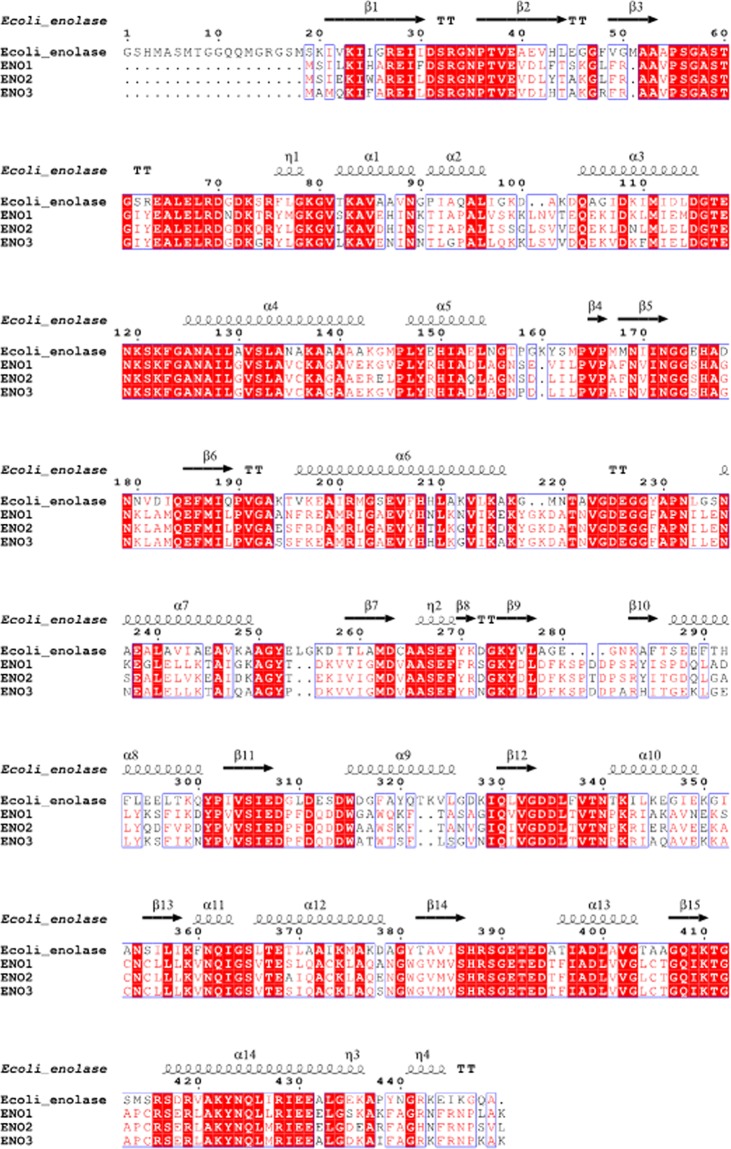
Sequence alignment of *Escherichia coli* and human enolase 1 (alpha), 2 (gamma) and 3 (beta) calculated with Clustal2 and visualized in ESpript3.0^46^.

Selectivity for the bacterial target over any human homologues is important in order to reduce the risk of adverse effects. A multiple sequence alignment using the primary sequences of *E. coli* enolase and the four known human isoforms indicates that the five enzymes share a percent identity ranging from 28–56% (see Fig. 6). While the catalytic residues are conserved across both species, there are significant variations in loop 2 and loop 3 that indicate selectivity may be achievable. Although our long-term strategy is focused on developing species-selective enolase inhibitors, it is important to note that Leonard *et al*., demonstrated that SF2312 was selectively cytotoxic towards *ENO1*-deleted glioma cells but displayed markedly reduced inhibition (GI_50_>200 µM) towards isogenic, *ENO1*-rescued cells^5^. A comparison of the crystal structures of SF2312 bound to human ENO2 and *E. coli* enolase show that the binding mode is nearly identical in both enzymes^5^. However, ENO2 residues Lys202 and Asp203 form an α-helix that is not present in bacterial enolase. In the dimer complex, these residues work in concert with loop 3, residues 248–272 in ENO2, to shift His158 approximately 1 Å compared to *E. coli* enolase. As the majority of amino acid variations occur in loops 2–3 and at the dimer interface, selectivity may be imparted through functionalization of C2 and C3 position, irrespectively of manipulation of the phosphate group (see Figs 1 and 6).

## Conclusion

The combination of MD simulations, crystal structures, biochemical and kinetic characterization, and antibacterial activity analysis has allowed us to elucidate the basis of *E. coli* enolase inhibition observed with SF2312 and its carbonyl substituted congener (KSF). Novel co-crystal structures of *E. coli* enolase bound with SF2312 and the KSF derivative indicate that SF2312 is an analogue to the carbanion reaction intermediate, and one minor chemical change can significantly disrupt protein-ligand interactions required for efficient inhibition. With a stronger and more stable attraction of the hydroxamic acid group to Mg1 and the orchestrated role of Mg2 in the release of the ligand from the bound state, as seen by the MD simulations, it is clear that coordination of both metal ions plays an important role in stabilizing and prolonging the interaction of SF2312 with the active site residues of enolase. Additionally, it was determined that the antibacterial activity of SF2312 relies on active transport across the bacterial cell envelope, and enolase inhibition was confirmed to be the primary mechanism of action through synergy studies with fosfomycin. Provided with a new permeability route due to the action of fosfomycin, the combinatorial therapy of SF2312 with fosfomycin offers a viable alternative to conventional monotherapy treatment against bacterial infections. We believe SF2312 represents an exciting and promising lead for further antibacterial drug development.

## Methods

### Steady-state kinetics

The steady-state kinetic parameters of K_M_ for 2-PGA, k_cat_, k_cat_/K_M_ and K_i_ were obtained for the purified recombinant *E. coli* enolase and compared to the enolase enzymes from other sources. Determination of K_M_ had also provided the k_cat_ and the catalytic efficiency values (k_cat_/K_M_). The standard reaction mixture contained 50 mM Tris pH 8.0, 50 mM KCl, 2 mM MgCl_2_ and enolase sample (2 μg). The 2-PGA concentrations varied from 0.0035 to 2 mM. The reaction was initiated by the addition of the substrate. Average of three independent experiments were used to obtain velocity values that were plotted as a function of 2-PGA concentration using GraphPad Prism8 software. The data was then fitted to the Michaelis-Menten equation.

### Enzymatic activity and inhibition assay

The activity and inhibition of *E. coli* enolase described in this study was carried out using the procedure as we previously reported^6^. Briefly, enolase activity was measured at 25 °C in the forward (formation of PEP from 2‐PGA) direction by direct monitoring of the increase in PEP absorbance at 240 nm, using a DU 640 spectrophotometer (Beckman). The standard assay contained 50 mM Tris pH 8.0, 0.1 M KCl, 0.5 mM 2-PGA and 1 mM MgSO_4_. The reactions were carried out at a final volume of 500 μL. Initial velocities came from the slopes of linear progress curves of 1 minute duration. One unit of enolase activity was defined as the amount of protein which catalyzes the formation of 1 μmole PEP from 2-PGA in 1 minute. The concentration of PEP was determined using a molar extinction coefficient (Ɛ_240nm_ = 1400 M^−1^ cm^1^).

Inhibition of enolase activity by SF2312 and its analog, KSF was performed at a constant enolase concentration of 40 nM. The compounds, supplied in water, were preincubated with protein at concentrations ranging from 2 nM to 20 µM for 5 minutes in the assay buffer consisting of 50 mM Tris pH 8.0, 0.1 M KCl and 1 mM MgSO_4_. The reaction was initiated by the addition of 0.5 mM 2-PGA. The decrease in enolase activity upon inhibition was monitored at 5 second intervals for a period of 5 minutes at λ =240 nm. The average half maximal inhibitory concentrations (IC_50_) was calculated from initial rates of absorbance increase plotted as a function of ligand concentration using GraphPad Prism software (Fig. 2). K_i_ values were calculated using the Cheng-Prusoff equation from the experimental IC_50_ and K_m_ values.

### Differential scanning calorimetry

The DCS experiment was performed as described previously^6^. Prior to analysis, purified *E. coli* enolase was buffer exchanged into 20 mM HEPES (pH 7.0), 50 mM NaCl and 2 mM MgCl_2_. The concentrations of protein and ligand were adjusted to 6 μM and 60 μM, respectively. Protein samples with and without ligand were heated from 0–120 °C at 1 °C/min using a NanoDSC instrument (TA instruments). The samples were degassed for 15 minutes prior to injection into the calorimeter cell. The reference cell was filled with buffer for all runs. A pressure of 3 atm was applied to both cells during the run. The excess heat capacity scans for the protein transitions were obtained by subtracting a control scan of buffer versus buffer. The data were corrected for the difference in heat capacity between the initial and the final state by using a sigmoid baseline in the NanoAnalyze software (TA instruments) and a two-state transition model was used to determine the denaturation temperature (T_m_) (Fig. 3).

### Antibacterial activity assays

To evaluate the activity of SF2312 against Gram-positive and Gram-negative bacteria, we have determined the minimal inhibitory concentration (MIC) for *Escherichia coli* ATCC 25922, *Staphylococcus aureus* ATCC 43000, *Pseudomonas aeruginosa* ATCC 27853, and *Acinetobacter baumannii* ATTC 19606 using the microdilution broth assay based on Clinical and Laboratory Standards Institute. Concentrations of SF2312 and the standard antibiotics tested were serially diluted to the following ranges: SF2312, 300 to 0.2 μg/mL; trimethoprim 10 to 0.01 μg/mL and fosfomycin, 20 to 0.040 μg/mL. For synergy studies, using the checkerboard method the following concentration range was used: fosfomycin 10 to 0.156 μg/mL and SF2312 100 to 0.05 μg/mL. The cation-adjusted Mueller-Hinton broth was supplemented with 25 μg/mL glucose-6-phosphate. Growth was monitored at Abs 600 nm. The MIC of each antimicrobial agent alone and in combination was defined as the lowest concentration that inhibited visible growth of the organism and were determined in duplicate.

The definition of synergy used in these studies was the fractional inhibitory concentration (FIC) index, represented by the formula FIC= (MIC of fosfomycin in combination/MIC of fosfomycin alone) + (MIC of SF2312 in combination/MIC of the SF2312 alone). A calculated FIC index of less than 0.5 represented a synergistic effect (i.e., total effect greater than the sum of the individual antibiotic effects), a value between 0.5–2 represented an additive effect (i.e. no additional contribution from including the second antibiotic, compared with when the first antibiotic. A value greater than 2 represented an antagonistic effect (i.e., total effect less than the sum of the individual effects)^26^.

### Crystal structures of *E. coli* enolase with SF2312 and KSF

Co-crystallization was used to obtain the ligand-enolase complex crystals. Based on the established crystallization conditions for *E. coli* enolase, the cleaved form of enolase was incubated at 12 mg/mL with ligand for a few hours on ice. An equal volume of the protein-ligand solution was then mixed with a reservoir solution containing 2.3 M ammonium sulfate, 0.2 M Na/K tartrate, 0.1 M MES (pH 6.0), and 2 mM MgCl_2_. High quality crystals appeared following incubation at 24 °C for one week. Crystals used for an overnight soaking experiment were transferred into an artificial mother liquor solution supplemented with 2 mM inhibitor and 5 mM MgCl_2_. Prior to freezing, crystals were cryoprotected with 25% (v/v) glycerol and flash cooled with liquid nitrogen.

Data collection was performed remotely at beam line 9–2 at the Stanford Synchrotron Radiation Laboratory (SSRL) using a Dectris Pilatus 6 M detector. Images were processed in CCP4i2 using iMosflm^28,29^. Molecular replacement was performed with the CCP4i2 (PHASER^30^) software using the substrate-bound structure of *E. coli* enolase as a model (PDB ID: 6BFY^6^). Six molecules (three dimers) were found in the asymmetric unit. Rebuilding and refinement were done in CCP4i2 using COOT^31^ and Refmac5^32^.

### Synthesis of SF2312 and KSF

The natural product SF2312 was synthesized according to a reported method^33^. This method was modified to synthesize the keto-analog (see Scheme 1 and Scheme S1). Compounds **1, 2, 3, 4** were synthesized analogous to a previously reported method^33^. Oxidation of **4** using pyridinium chlorochromate (PCC) in the presence of molecular sieves provides diethyl (1-(benzyloxy)-2,5-dioxopyrrolidin-3-yl)phosphonate **5** in quantitative yield. Cleavage of the phosphonic ester was achieved by trimethylsilyl iodide and then hydrogenolysis to afford the desired product (1-hydroxy-2,5-dioxopyrrolidin-3-yl)phosphonic acid **7**. Detailed syntheses for compounds **1–7** are provided in the Supplementary Materials (see Scheme S1).

Analytical thin-layer chromatography was performed using glass plates precoated with 200−300 mesh silica gel impregnated with a fluorescent indicator (254 nm). NMR spectra were recorded in CDCl_3_, MeOH-d_4_ on Bruker NMR (500 MHz) with TMS as an internal reference. ^31^P NMR spectra was referenced against H_3_PO_4_. Preparatory HPLC was conducted using LC Shimadzu (LC-20AP) and Phenomenex column C_18_ 250 × 21 mm (4 μm).

### Metadynamics simulations

Systems were initially equilibrated first for 100 ns in the NPT ensemble, during which no significant changes in the protein (RMSD <1.7 Å) and the ligand (RMSD <0.25 Å) are observed. The equilibrium KSF simulations were initiated from chain F monomer of the enolase:KSF crystal structure, while the equilibrium SF2312 simulations were initiated from the chain A monomer of the enolase:SF2312 crystal structure. Missing residues in enolase of the enolase:KSF crystal structure were built-in by aligning to the enolase:SF2312 structure and copying in the coordinates from the SF2312 system. The systems were solvated with the TIP3P water model and 150 mM NaCl, and protonation states of the amino acid residues were based upon model system pK_a_s at pH 7. The ligand parameters were obtained using CGenFF^34,35^ (https://cgenff.umaryland.edu) and CHARMM36m force field^36^ was used for the protein and solvent components. All simulations were performed using the GROMACS 2018 program^37^. Energy minimization and equilibration steps were performed according to the CHARMM-GUI protocol, running 500 steps of steepest descent minimization and six steps of canonical ensemble equilibrations for 25 ps each with 1 fs timestep. Positional restraints were applied on the heavy atoms, gradually decreasing the force constants after every equilibration step. The systems were run again for 100 ns using a timestep of 2 fs. Temperature was maintained at 300 K using the Nose-Hoover thermostat^38,39^ with coupling time constant of 1 ps. Pressure was maintained at 1 bar through isotropic pressure coupling with a coupling constant of 5 ps, using the Parrinello-Rahman barostat^40^. Van der Waals interactions were cut off at 12 Å, with interactions modified using the force-switch method between 10 and 12 Å. Long-range electrostatic interactions were calculated using the particle mesh Ewald method, with real-space cutoff of 1.2 nm.

Metadynamics simulations were initiated from the final structure of each equilibrated system. We performed metadynamics using the frequency-adaptive metadynamics scheme^41^ from the open-source, community-developed PLUMED library, version 2.5^42^. In order to carry out a successful transition between bound state and unbound state in all systems, a suitable collective variable (CV) must be carefully chosen. In our systems, the collective variable is the distance between the COM of the ligand and the COM of six residues on the β-barrel of the protein. The β-barrel COM coordinate was defined by the side chain heavy atoms of residues Glu167, Asp245, Glu289, Asp316, Lys341, and His369 (Fig. 5A). Ten independent simulations each of the KSF and SF2312 without Mg2 systems, while eleven independent simulations of the SF2312 with Mg2 system exhibited successful unbinding of the ligand, which were used for further analyses. The intermittently added Gaussian functions added to carry out frequency-adaptive metadynamics is stored in a file called HILLS. In this file, the CV distance every 10 ps is reported. Because the unbinding time of the ligand varies in each independent simulation, we chose 7.0 nm as a safe CV distance to consider the ligand to be in the unbound state. Thus, we trimmed each HILLS file such that the last datapoint is when the ligand first crosses the 7.0 nm CV distance. There are some simulations where the ligand did not reach the 7.0 nm CV distance (lowest is 5.47 nm), but regardless, the ligand has still reached the unbound state in those instances. This is then used as an input to a post-processing method called sum_hills to reconstruct the free energy function. To assess reliability of the calculated metadynamics unbinding time distribution and to determine the characteristic unbinding times for each system, a two-sample Kolmogorov-Smirnov (KS) test^43^ was performed *a posteriori* as implemented in Matlab R2018a^44,45^. This test determines whether the empirical cumulative distribution function obtained from calculations is similar to a theoretical Poisson cumulative distribution function^41,43^.

## Supplementary information

Supplementary information
