# Supplementary material for: Functional and structural basis of E. coli enolase inhibition by SF2312: a mimic of the carbanion intermediate
Source: Sci Rep. 2019 Nov 19;9:17106. doi: 10.1038/s41598-019-53301-3 (PMC6863902; doi:10.1038/s41598-019-53301-3)
Supplement: Supplementary file 1 — Supplementary information [file 41598_2019_53301_MOESM1_ESM.pdf]

# Functional and structural basis of *E.coli* enolase inhibition by SF2312: a mimic of the carbanion intermediate.

Jolanta Krucinska,[†,⊥] Michael N. Lombardo,[†,⊥] Heidi Erlandsen,[‡,⊥] Akram Hazeen,[‖] Searle S. Duay[‖], Jason G. Pattis[§], Victoria L. Robinson[§], Eric R. May[§] and Dennis L. Wright[*,†,‖]

† Department of Pharmaceutical Sciences, University of Connecticut, 69 North Eagleville Road, Storrs, Connecticut 06269, United States

‡ Center for Open Research Resources & Equipment (COR2E), University of Connecticut, 91 North Eagleville Road, Storrs, Connecticut 06269, United States

§ Department of Molecular and Cellular Biology, University of Connecticut, 91 North Eagleville Road, Storrs, Connecticut 06269, United States

‖ Department of Chemistry, University of Connecticut, 55 North Eagleville Road, Storrs, Connecticut 06269, United States

⊥These authors contributed equally to this work

*Corresponding author: dennis.wright@uconn.edu

**SUPPLEMENTARY INFORMATION:**

**Scheme S1**: Synthetic route for SF2312-analog (KSF).

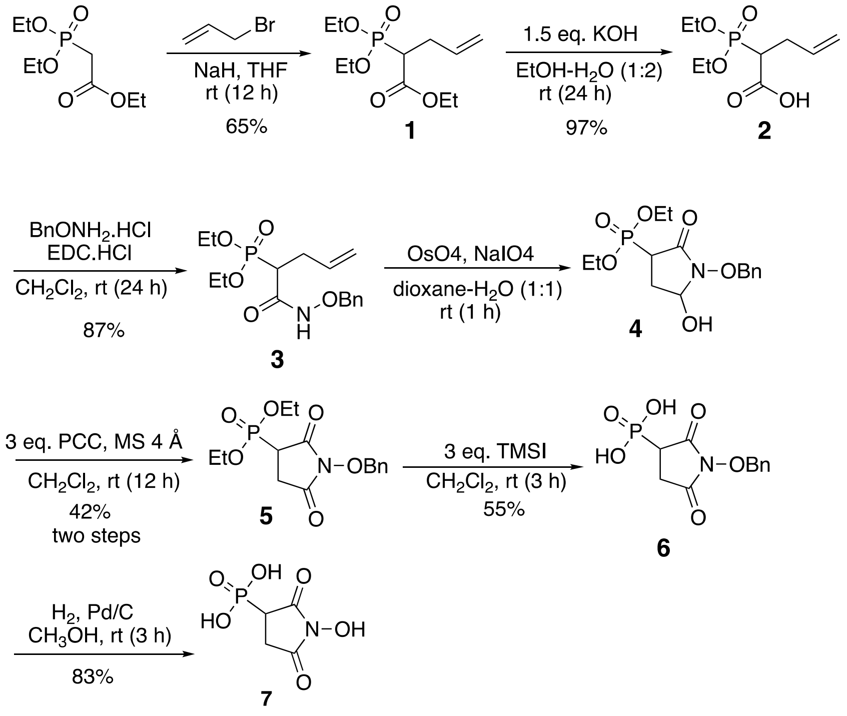

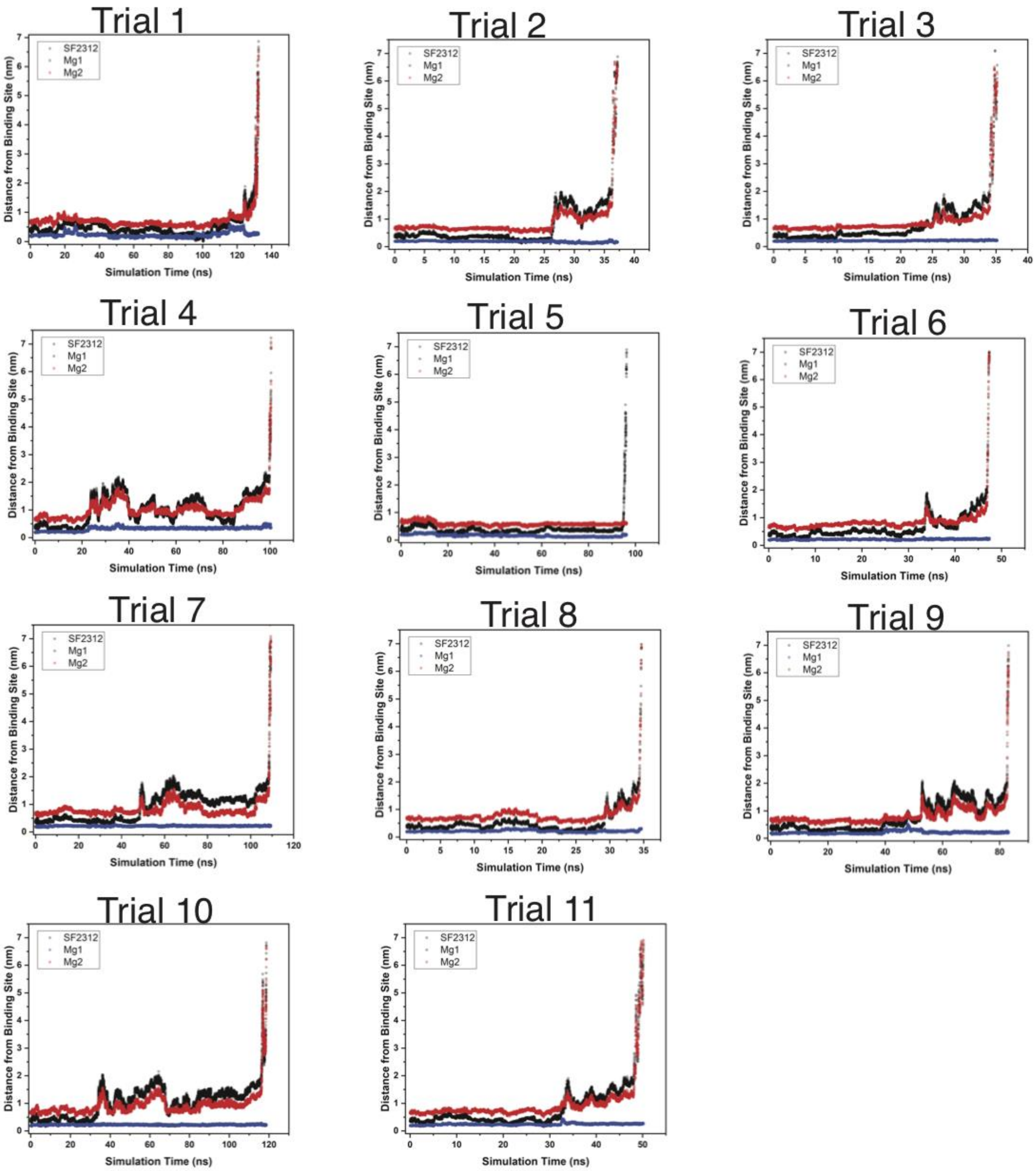


**Figure S1.** Distances between the binding site COM and the inhibitor COM, MG1 and Mg2 over the time course of the eleven metadynamics trajectories for SF2312 with both Mg ions. In all trials, except trail 5, SF2312 and MG2 positions are highly correlated, indicating they are strongly interacting through the inhibitor dissociation pathways.

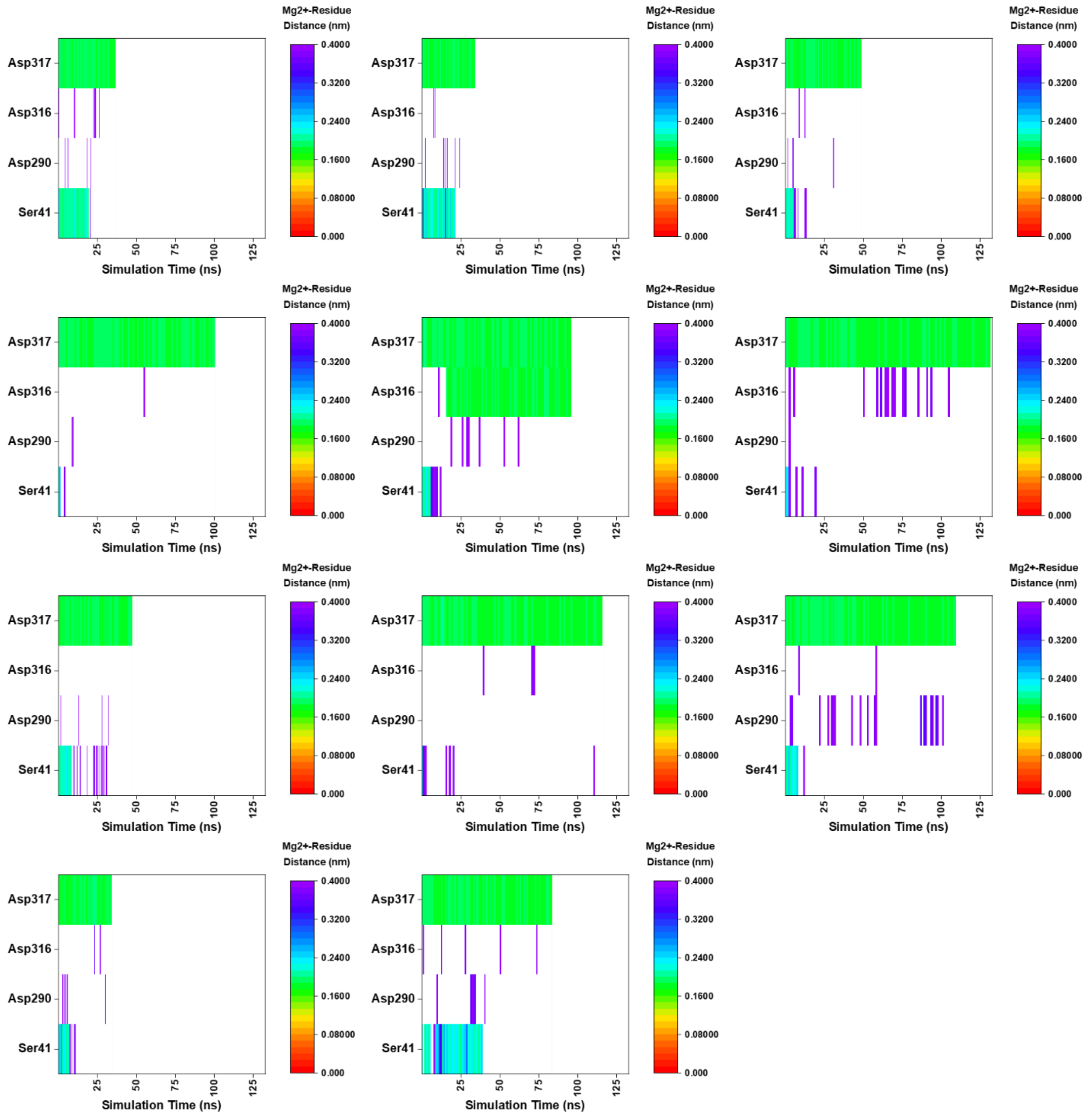


**Figure S2.** Distances of residues that are within 4 Å radius of Mg2 on each trial. The residues are Ser41, Asp290, Asp316, and Asp317. A heat map from red, indicating 0.0 nm distance, to purple, indicating 0.4 nm distance, was used. Absence of color means that the residue is not within 4 Å radius of Mg2. Asp317 is the last residue to be in contact with Mg2 before SF2312 completely unbinds.

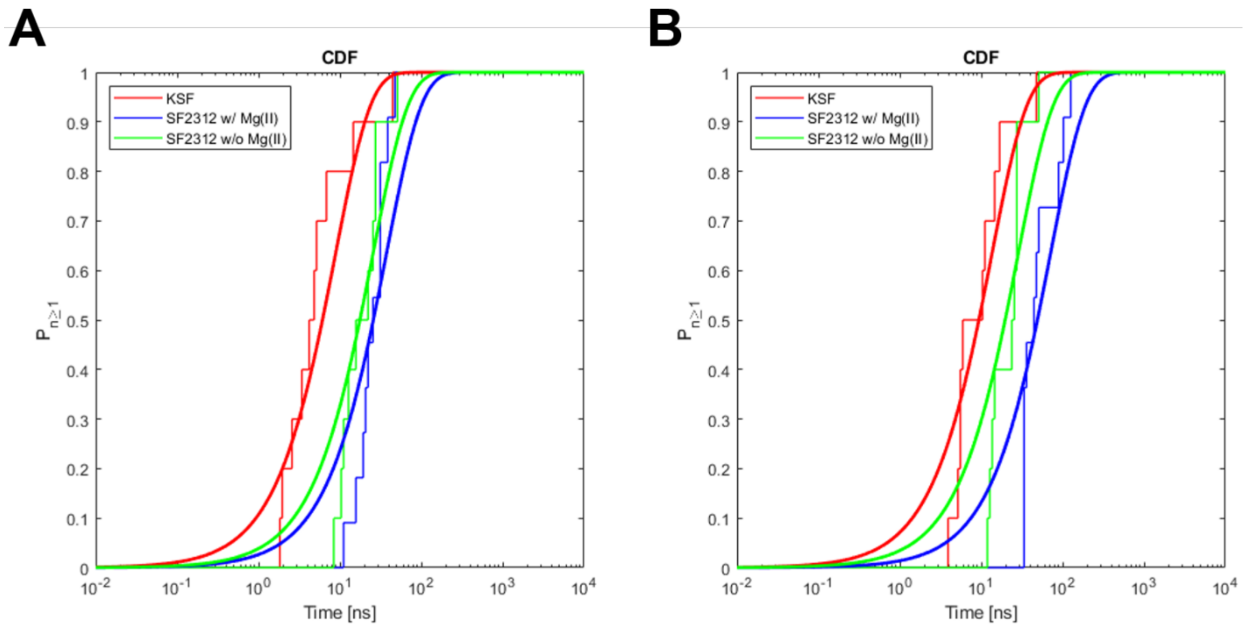


**Figure S3.** Results of the Kolmogorov-Smirnov (KS) test from the transition times of the first energy barrier (CV = 0.7 nm) (A) and second energy barrier (CV = 2.1 nm) (B). Probability of observing at least one transition by time *t* evaluated from the simulations (thin lines) and fitted as a Poisson process (thick lines).

**Table S1.** Simulations of the transition between bound and unbound states of the ligand. Mean transition time μ, standard deviation σ, and characteristic time τ are reported with the μ/σ and (μ ln 2)/$t_m$ ratios. The p-values from the KS statistics are also reported and p-value of less than 0.05 indicates significant difference between empirical cumulative distribution function (CDF) and theoretical CDF.

| | **1st Energy Barrier** | | | **2nd Energy Barrier** | | |
|---|---|---|---|---|---|---|
| | **KSF** | **SF2312 w/ Mg2** | **SF2312 w/o Mg2** | **KSF** | **SF2312 w/ Mg2** | **SF2312 w/o Mg2** |
| μ (ns) | 9 ± 4 | 28 ± 3 | 22 ± 4 | 13 ± 4 | 60 ± 10 | 25 ± 4 |
| σ (ns) | 13 | 11 | 13 | 13 | 35 | 12 |
| $t_m$ (ns) | 5 | 26 | 20 | 8 | 47 | 25 |
| μ/σ | 0.69 | 2.55 | 1.64 | 1.00 | 1.72 | 1.98 |
| (μ ln 2)/$t_m$ | 1.40 | 0.72 | 0.77 | 1.07 | 0.88 | 0.67 |
| τ (ns) | 8.8 | 37.2 | 25.4 | 13.5 | 70.7 | 28.4 |
| p-value | 0.475 | 0.338 | 0.298 | 0.419 | 0.060 | 0.132 |

## CHEMISTRY

The natural product SF2312 was synthesized according to a reported method[46]. This method was modified in order to synthesize the analog for this compound (Scheme S1). Compounds **1, 2, 3, 4** were synthesized analogous to a previously reported method[46]. Oxidation of **4** using pyridinium chlorochromate (PCC) in the presence of molecular sieves provides diethyl (1-(benzyloxy)-2,5-dioxopyrrolidin-3-yl)phosphonate **5** in quantitative yield. Cleavage of the phosphonic ester was achieved by trimethylsilyl iodide and then hydrogenolysis to afford the desired product (1-hydroxy-2,5-dioxopyrrolidin-3-yl)phosphonic acid **6**.

***Ethyl 2-(diethoxyphosphoryl)pent-4-enoate (1):*** Triethyl phosphonate (1.01 g, 4.51 mmol) was dissolved in THF (10 mL) and cooled in an ice bath. To this solution was added NaH 60% (197 mg, 4.92 mmol, 1.09 eq.) and let stir at 0 °C for 1h. Then, allyl bromide (0.42 mL, 4.85 mmol, 1.08 eq.) was added and the solution was warmed to room temperature and left stirring overnight. Next day, the reaction was quenched with saturated solution of ammonium chloride (5 mL) and diluted with $H_2O$ (10 mL). It was extracted with $CHCl_3$ (3 × 20 mL), and then the combined organics were washed with brine (20 mL), dried over $Na_2SO_4$ and solvent was removed under vacuum resulting in pale yellow oil. It was purified by prep. HPLC using gradient elution with a mobile phase $H_2O/CH_3CN$ (90/10 to 5/95%) to afford the title compound as a colorless oil (780 mg, 65%). $R_f$ value 0.6 (EtOAc); IR (neat) ν max: 2982, 1732, 1240, 1155, 1017, 961 $cm^{-1}$; $^1H$ NMR ($CDCl_3$, 500 MHz) δ (ppm) 5.76 (m, 1H), 5.12 (dd, *J* = 17.1, 1.5 Hz, 1H), 5.05 (dd, *J* = 10.2, 1.4 Hz, 1H), 4.16 (m, 6H), 3.03 (ddd, *J* = 22.4, 11.2, 3.8 Hz, 1H), 2.71 (m, 1H), 2.59 (m, 1H), 1.34 (td, *J* = 7.1, 3.7 Hz, 6H), 1.28 (t, *J* = 7.1 Hz, 3H); $^{13}C$ NMR ($CDCl_3$, 125 MHz) δ (ppm) 168.6 (d, *J* = 5 Hz), 134.6 (d, *J* = 16.2 Hz), 117.13, 62.8 (d, *J* = 16.2) 62.7 (d, *J* = 6.2), 61.38, 45.96, 44.93, 31.0 (d, *J* = 3.8 Hz), 16.4 (dd, *J* = 5, 2.5Hz), 14.17; $^{31}P$ NMR ($CDCl_3$, 200 MHz) δ (ppm) 22.6; HRMS (ESI) (m/z) calcd for $[C_{11}H_{21}O_5P + Na]^+$, 287.1013, observed 287.0935.

***2-(Diethoxyphosphoryl)pent-4-enoic acid (2)****:* Compound **1** (630 mg, 2.38 mmol) were dissolved in ethanol (5.0 mL). Then 2 N KOH (1.8 mL, 3.6 mmol, 1.5 eq.) was added and the solution was stirred overnight. The sample was diluted with $H_2O$ (10 mL) and washed with $CH_2Cl_2$ (2 × 20 mL). It was then acidified with 2 N HCl to pH = 2 and extracted with $CH_2Cl_2$ (3 × 20 mL), combined organics were washed with brine, dried over $Na_2SO_4$ and with a rotavapor resulting in colorless oil (544 mg, 97% yield). $R_f$ value 0.31 (EtOAc/MeOH = 90:10); IR (neat) ν max: 2983, 1727, 1217, 1160, 1014 $cm^{-1}$; $^1H$ NMR ($CDCl_3$, 500 MHz) δ (ppm) 8.90 (sb, 1H), 5.79 (m, 1H), 5.14 (dd, *J* = 17.0, 1.7 Hz, 1H), 5.06 (d, *J* = 10.1 Hz, 1H), 4.20 (m, 4H), 3.06 (ddd, *J* = 22.7, 10.9, 3.9 Hz, 1H), 2.70 (m, 1H), 2.54 (m, 1H), 1.35 (td, *J* = 7.1, 3.2 Hz, 6H); $^{13}C$ NMR ($CDCl_3$, 125 MHz) δ (ppm) 170.4 (d, *J* = 3.8 Hz), 134.4 (d, *J* = 15 Hz), 117.2, 63.7 (d, *J* = 6.2 Hz), 63.0 (d, *J* = 7.5 Hz), 45.8, 44.8, 30.9 (d, *J* = 5 Hz), 16.3 (d, 6.2 Hz); $^{31}P$ NMR ($CDCl_3$, 200 MHz) δ (ppm) 23.6; HRMS (ESI) (m/z) calculated for $[C_9H_{17}O_5P + H]^+$, 237.0881, observed 237.0809.

***Diethyl (1-((benzyloxy)amino)-1-oxopent-4-en-2-yl) phosphonate*** *(3):* Compound **2** (644 mg, 2.73 mmol) and O-benzyloxy hydroxylamine hydrochloride (530 mg, 3.32 mmol, 1.2 eq.) were dissolved in $CH_2Cl_2$ (25 mL). DMAP (1.07 g, 8.76 mmol, 3 eq.) and EDC.HCl (631 mg, 3. 29 mmol, 1.2 eq.) were added and the solution was stirred for 36 h at room temperature. It was then diluted with $CH_2Cl_2$ (25 mL) and washed with 1 N HCl (2 × 25 mL), brine (25 mL), dried over $Na_2SO_4$ and with a rotavapor resulting in pale yellow oil (815 mg, 87% yield). $R_f$ value 0.31 (EtOAc); IR (neat) ν max: 3175, 2981, 1690, 1668, 1226, 1017 $cm^{-1}$; $^1H$ NMR ($CDCl_3$, 500 MHz) δ (ppm) 9.29 (s, 1H), 7.40-7.30 (m, 5H) , 5.75 (m, 1H), 5.11 (dd, *J* = 17.0, 1.5 Hz, 1H), 5.04 (dd, *J* = 10.2, 1.5 Hz, 1H), 4.91 (s, 2H), 4.12 (m, 4H), 2.71 (m, 2H), 2.52 (m, 1H), 1.31 (t, *J* = 7.1 Hz, 6H); $^{13}C$ NMR ($CDCl_3$, 125 MHz) δ (ppm) 165.3, 135.3, 134.4 (d, *J* = 13.8 Hz), 129.2, 128.6, 128.5, 117.4, 78.3, 63.3 (d, *J* = 6.2 Hz), 62.8 (d, *J* = 6.2 Hz), 43.7 (d, 130 Hz), 30.7, 16.3 (dd, *J* =

6.2, 3.8 Hz); $^{31}P$ NMR ($CDCl_3$, 200 MHz) δ (ppm) 24.3; HRMS (ESI) (m/z) calculated for $[C_{16}H_{12}NO_5P + C_{16}H_{12}NO_5P + Na]^+$, 705.2665, observed 705.2709.

***Diethyl (1-(benzyloxy)-2,5-dioxopyrrolidin-3-yl) phosphonate** (5):* compound **3** (509 mg, 1.49 mmol) was dissolved in dioxane/$H_2O$-1:1 (25 mL). Then, a solution of aqueous $OsO_4$ 4% (474 μL, 74.6 μmol, 5 mol%) was added and let stir for 30 min at room temp. To this solution, $NaIO_4$ (957 mg, 4.47 mmol, 3 eq.) was added in portions and left stirring for 1 h. It was then diluted with $H_2O$ (20 mL) and extracted with $CH_2Cl_2$ (3 × 20 mL), washed with brine (25 mL), dried over $Na_2SO_4$ and solvent was removed under vacuum resulting in yellow oil. It was purified by silica gel using ($CHCl_3$/MeOH = 9:1) resulting in yellow oil (277 mg, 54% yield) and used for next step. This intermediate (167 mg, 0.486 mmol) was dissolved in $CH_2Cl_2$ (3.0 mL). Molecular Sieves 4 Å (100 mg) and PCC (316 mg, 1.46 mmol, 3 eq.) were added and the mixture left stirring at room temp. overnight. It was then filtered through a pad of celite and purified by silica gel containing 30% cysteine and eluted with 100% EtOAc to yield a yellow (130 mg, 78% yield). $R_f$ value 0.68 (EtOAc/MeOH =100:10); IR (neat) ν max: 2983, 1788, 1722, 1209, 1013 $cm^{-1}$; $^1H$ NMR ($CDCl_3$, 500 MHz) δ (ppm) 7.50 (dd, $J$ = 6.6, 2.9 Hz, 2H), 7.40 – 7.35 (m, 3H), 4.29 – 4.13 (m, 4H), 3.21 (dd, $J$ = 10, 5 Hz, 1H), 2.92 (m, 2H), 1.37 (t, $J$ = 7.0 Hz, 3H), 1.34 (t, $J$ = 7.0 Hz, 3H); $^{13}C$ NMR ($CDCl_3$, 125 MHz) δ (ppm) 169.1 (d, $J$ = 5 Hz), 166.6 (d, $J$ = 6.2 Hz), 133.2, 129.9, 129.4, 128.6, 78.9, 63.7 (dd, $J$ = 63.8, 7.5 Hz), 36.7 (d, $J$ = 16.2 Hz), 27.9 (d, $J$ = 3.8 Hz), 16.4 (t, $J$ = 5 Hz); $^{31}P$ NMR ($CDCl_3$, 200 MHz) δ (ppm) 19.6; HRMS (ESI) (m/z) calculated for $[C_{15}H_{20}NO_6P + H]^+$, 342.1096, observed 342.0992.

***(1-(benzyloxy)-2,5-dioxopyrrolidin-3-yl) phosphonic acid** (6):* compound **5** (125 mg, 0.366 mmol) was dissolved in dry $CH_2Cl_2$ (3.0 mL) and TMSI (156 μL, 1.10 mmol, 3 eq.) was added. It was stirred at room temperature overnight and then solvent was removed under vacuum. The residue was purified by prep HPLC resulting in colorless oil (52 mg, 50% yield). $R_f$ value 0.54

(MeOH/$H_2O$ = 80:20); IR (neat) ν max: 1787, 1704, 1391, 1206 $cm^{-1}$; $^1H$ NMR (MeOH-$d_4$, 500 MHz) δ (ppm) 7.49 (dd, $J$ = 6.7, 2.9 Hz, 2H), 7.39 – 7.34 (m, 3H), 5.04 (s, 2H), 3.36 (ddd, $J$ = 22.6, 9.5, 3.5 Hz, 1H), 2.99 (dt, $J$ = 18.0, 10.0 Hz, 1H), 2.82 (td, $J$ = 18.3, 3.5 Hz, 1H); $^{13}C$ NMR (MeOH-$d_4$, 125 MHz) δ (ppm) 171.9 (d, $J$ = 4.6 Hz), 169.9 (d, $J$ = 5.6 Hz), 135.2, 131.0, 130.3, 129.6, 79.8, 39.0 (d, $J$ = 135.9 Hz), 29.3 (d, $J$ = 3.6 Hz); $^{31}P$ NMR ($CDCl_3$, 200 MHz) δ (ppm) 17.6.

***(1-hydroxy-2,5-dioxopyrrolidin-3-yl) phosphonic acid** (7):* compound **6** (38 mg, 0.133 mmol) was transferred to a vial equipped with a stir bar. It was dissolved in MeOH (2 mL) and bubbled with Ar for 10 min followed by the addition of Pd/C (10 mg). It was subjected for hydrogenolysis conditions. After 1 h, it was filtered through a plug of $C_{18}$ and with a rotavapor resulting in colorless oil (22 mg, 84% yield). $R_f$ value 0.48 (MeOH/$H_2O$ = 60:40); IR (neat) ν max: 1780, 1689, 1221 $cm^{-1}$; $^1H$ NMR ( $D_2O$, 500 MHz) δ (ppm) 3.31 (ddd, $J$ = 21.9, 9.1, 3.1 Hz, 1H), 3.05 (dt, $J$ = 18.5, 9.2 Hz, 1H), 2.85 (td, $J$ = 17.0, 3.0 Hz, 1H); $^{13}C$ NMR ($D_2O$, 125 MHz) δ (ppm); $^{31}P$ NMR ( $D_2O$, 200 MHz) δ (ppm) 14.1; HRMS (ESI) (m/z) calculated for $[C_4H_6NO_6P + Na]^+$, 217.9819, observed 217.9781.
